# Outcomes and Effects of 250-cm Biliopancreatic Limb One Anastomosis Gastric Bypass in Patients with BMI > 50 kg/m^2^ with Total Bowel Length > 6 m: a 2-Year Follow-up

**DOI:** 10.1007/s11695-022-06078-w

**Published:** 2022-04-29

**Authors:** Moheb S. Eskandaros

**Affiliations:** Department of General Surgery, Faculty of Medicine, Ain Shams University, Cairo, Egypt

**Keywords:** One anastomosis gastric bypass, BMI > 50, Long biliopancreatic limb, Total bowel length

## Abstract

**Background:**

There is a controversy on the suitable bariatric procedure for patients with BMI > 50 kg/m^2^. Many surgeons prefer the Roux en-Y gastric bypass (RYGB) while others resort to long biliopancreatic limb (BPL) one anastomosis gastric bypass (OAGB).

**Methods:**

This study included patients with BMI > 50 kg/m^2^ who underwent 250-cm BPL OAGB with total bowel length (TBL) > 6 m and completed 24-month follow-up from July 2015 to November 2021. Demographic data with preoperative weight, BMI, hypertension (HTN), DM (HbA1C), Hb, iron, calcium, albumin, vitamin D, and parathormone levels (PTH) were recorded. Operative time, total bowel length (TBL), residual length (RBL), complications and postoperative weight, BMI, % of total weight loss (%TWL), HTN, DM, and alkaline reflux as well as Hb, iron, calcium, albumin, vitamin D, and PTH levels were recorded at 6, 12, 18, and 24 months.

**Results:**

OAGB had a significant decrease in weight, BMI (25.6 ± 3.4 kg/m^2^ at 24 months) with %TWL of 48 ± 5% at 24 months. TBL and RBL were 6.7 ± 0.65 and 4.2 ± 0.65 m respectively. %BL (RBL/TBL*100) was 62.4 ± 3.3%. The HbA1C, HTN, and alkaline reflux incidence were 5.5 ± 0.29 gm%, 1.4%, and 3.7% respectively at 24 months. Hb, iron, calcium, albumin, and vitamin D showed a significant decrease but still within normal range, and PTH showed a significant increase at 24 months.

**Conclusion:**

Long BP (250 cm) OAGB in patients with BMI > 50 kg/m^2^ with TWL > 6 m had good results in the achievement of weight loss and weight maintenance goals with remission of associated comorbidities as HTN and DM.

**Graphical abstract:**

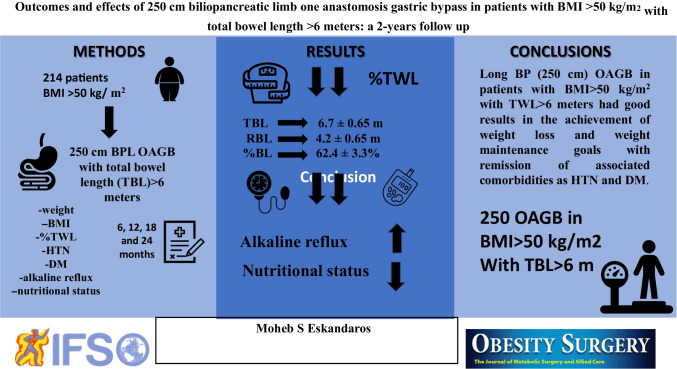

## Introduction

The ideal bariatric procedure that provides the ideal postoperative body mass index (BMI) [1] with the remission of associated medical conditions as diabetes mellitus (DM) and hypertension (HTN) at minimal postoperative nutritional deficiencies and complications has not been found yet [2]. Many bariatric procedures are popular nowadays as sleeve gastrectomy (SG), Roux-en-Y gastric bypass (RYGB), and one anastomosis gastric bypass (OAGB) [3], and each one has its advantages and disadvantages. As a result, there is a controversy in the choice of the suitable procedure for each patient especially patients with body mass index (BMI) > 50 kg/m^2^ [4–6]. This class of patients usually resort to procedures that may fail to achieve target BMI [7] and require redo operations [8] or those that achieve the target BMI but exposes the patient to a debilitating course of nutritional deficiencies and malnutrition that may require reversal/redo operations or cause mortality. This may be due to subjective differences especially the total bowel length (TBL) and the absorptive capacity of the residual functioning part of the gastrointestinal tract.

The classical procedure of choice by most bariatric surgeons for patients with a BMI > 50 kg/m^2^ is the RYGB despite its technical difficulties and complications [9, 10]. OAGB has been introduced as an easy-to-perform one-stage procedure, and bariatric surgeons modified it to suit patients with BMI > 50 kg/m^2^ by increasing the length of the biliopancreatic limb (BPL) to accomplish more weight loss. However, the results obtained were not always the same. The YOMEGA study compared the 200-cm BPL OAGB with 150-cm alimentary limb (AL) and 50-cm BPL RYGB in patients with BMI > 40 kg/m^2^ or with BMI > 35 kg/m^2^ with associated medical conditions as DM, HTN, dyslipidemia, obstructive sleep apnea, or arthritis [11].

Yet, the application of long BPL OAGB in patients with BMI > 50 kg/ m^2^ has not been thoroughly studied in correlation with the TBL. In addition, the application of 250 cm in OAGB was not applied by many surgeons for fear of the nutritional drawbacks.

This study was different from the YOMEGA study in evaluating a 250-cm BPL OAGB (longer than 200 cm in YOMEGA) in patients with BMI > 50 kg/m^2^ (higher BMI than YOMEGA) specifically with more than 6-m TBL as a potential solution for obtaining the target BMI without causing morbidity for those patients with BMI > 50 kg/m^2^ guided by the TBL.

## Patients and Methods

This study included 214 patients with BMI > 50 kg/m^2^ who underwent long BPL OAGB (250 cm) with TBL being measured (and found to be > 6 m) and completed 24-month follow-up from May 2015 to November 2021 in the department of general and bariatric surgery, Faculty of Medicine, Ain Shams University, Cairo, Egypt.

Inclusion criteria were patients between 18 and 60 years that underwent a primary OAGB, TBL was more than 6 m, not suffering from preoperative GERD symptoms, fit for surgery, and with no history of abdominal surgeries.

Exclusion criteria were previous bariatric (or operations other than the specified lengths) or abdominal surgeries, patients with hiatus hernia or GERD on routine preoperative upper GI endoscopy, or with cardiovascular or chest problems.

### Data Collection

Age, sex, height, weight, BMI, and associated medical conditions as DM (level of HbA1C), and HTN status where HTN was present with blood pressure > 140/90 mmHg were recorded preoperatively. Levels of hemoglobin (Hb), iron (Fe), calcium (Ca), albumin (Alb), vitamin D (Vit D), and parathormone (PTH) were recorded preoperatively.

The operative time and the incidence of postoperative complications were monitored. Total bowel length (TBL) was measured and recorded together with the residual bowel length (RBL) after bypassing 250 cm of proximal jejunum. Ratio (%BL) between TBL and RBL (RBL/TBL*100) representing the % of the remaining bowel length acting as the alimentary limb was calculated.

For each patient, the postoperative weight, BMI, percent of total weight loss (%TWL), the incidence of remission in preoperative medical conditions as HTN (defined as blood pressure below 140/90 mmHg without antihypertensive medications), DM (monitored through the change of level of HbA1C with level below 6 gm% without the use of antidiabetic drugs and insulin was considered as remission), incidence of alkaline reflux (by pH monitoring), and levels of Hb, Fe, Ca, Alb, Vit D, and PTH at 6, 12, 18, and 24 months were recorded. Patients underwent regular upper endoscopy at 6 monthly intervals to detect marginal ulcers and alkaline gastritis.

### Preoperative Investigations

All patients underwent routine preoperative investigations including routine laboratory investigations, chest X-ray, pulmonary function test, ECG, echocardiography, and upper GI endoscopy.

### Surgical Procedure

The gastric pouch was constructed by creating a window in the lesser omentum with a harmonic scalpel at the crow’s foot and firing a 45-mm cartridge horizontally and 4–5 cartridges vertically guided by a 36 F bougie for pouch calibration. The gastrojejunostomy was constructed at a distance of 250 cm from the duodenojejunal (DJ) junction by a 45-mm cartridge after measuring the entire length of the small intestine to make sure that TBL > 6 m. A minimum of 350 cm of the small intestine was ensured to be present distally to prevent the incidence of a short common limb (CL). Shorter lengths of the small intestine were allowed to have the RYGB procedure to be carried out instead and not included in the study. The enterotomies were then closed using V-lock sutures (V-lock 3/0, Medtronic™, Minneapolis, USA). The afferent and efferent loops were then fixed to the remnant stomach and the pylorus respectively using non-absorbable sutures to minimize reflux and facilitate the passage of food. An 18 F drain was left in the surgical bed.

### Data Analysis

The collected data were revised, coded, tabulated, and introduced to a PC using the Statistical Package for Social Science (SPSS 26). Data were presented, and suitable analysis was done according to the type of data obtained for each parameter. Student’s *t* test was used in continuous values. Pearson’s chi-squared test and Fisher’s exact test were used in categorical variables. Repeated measure ANOVA was done to detect the significance of the difference between means measured more than twice for the same study group. Pairwise comparisons were done within each pair of successive readings of variables using paired *t* test with a correlation between them. Scatter plots and Pearson product-moment bivariate correlation were done to determine the association between two variables.

## Results

From May 2015 to November 2021, 214 patients with BMI > 50 kg/m^2^ and had TBL > 6 m underwent 250-cm BPL OAGB who met the inclusion criteria and completed 24-month follow-up with 6-month interval.

### Preoperative and Operative Data

The preoperative age, height, weight, BMI, level of HbA1C, Hb, Fe, Ca, Alb, Vit D, PTH, operative time, TBL, RBL, %BL sex distribution, and HTN status were detailed in (Table 1). The mean DM duration was 5.7 ± 2.3 years. The mean TBL, RBL, and %BL were 6.7 ± 0.65 m, 4.2 ± 0.65 m, and 62.4 ± 3.3%.

**Table 1:** Preoperative age, height, weight, BMI, level of HbA1C, Hb, Fe, Ca, Alb, Vit D, PTH, operative time, TBL, RBL, %BL, sex distribution, and HTN status

OAGB (*n* = 214) Preoperative		Range		Mean^*^	SD
		Minimum	Maximum		
Age		22	57	38.6	10.1
Height		1.58	1.76	1.6	0.05
Weight		135	177	154.8	9
BMI		50	66.6	55.5	2.9
DM (HbA1C)		6.3	9.4	7.7	0.84
Hb (12–16 gm%)		11.8	14.9	13.3	0.86
Fe (60–180 mcg/dl)		142	181	162.6	10.5
Ca (8.6–10.6 mg/dl)		8.5	10.4	9.5	0.5
Albumin (3.5–4.5 gm%)		3.8	4.5	4.1	0.2
Vitamin D (20–50 ng/ml)		30	49	39.4	5.4
PTH (10–55 pg/ml)		26	50	38	7.1
Operative time		94	144	117	14
Total bowel length (TBL)		6.05	9.55	6.7	0.65
Residual bowel length (RBL)		3.55	7.05	4.2	0.65
%BL (RBL/TBL*100)		58.6	73.8	62.4	3.3
		*N*		%	
Sex	Female	150		70.1%	
	Male	64		29.9%	
HTN	No	82		38.3%	
	Yes	132		61.7%	

^*^Skewness value was between 1 and − 1 so mean ± SD was used

### Postoperative Data

The postoperative weight, BMI, %TWL, level of HbA1C, Hb, Fe, Ca, Alb, Vit D, PTH, alkaline reflux incidence, and HTN status at 6, 12, 18, and 24 months were detailed in (Table 2). The rate of complications was 5.1% (11/214 patients) with 3 patients having a minor anastomotic leak, 3 patients having DVT, 2 patients having hematomas, 2 patients having marginal ulcers, and 1 patient having a chest infection (pneumococcal pneumonia).

**Table 2:** Postoperative weight, BMI, %TWL, level of HbA1C, Hb, Fe, Ca, Alb, Vit D, PTH, alkaline reflux incidence, and HTN status at 6, 12, 18, and 24 months

OAGB (*n* = 214)	Time of measurement	Range		Mean^*^	SD
		Minimum	Maximum		
Weight	6 months	104	153	124.7	10.4
	12 months	75	128	99.2	11.2
	18 months	62	118	88.5	11
	24 months	43	100	71.5	10.8
BMI	6 months	38.2	56.2	44.7	3.2
	12 months	27.5	47.6	35.5	3.5
	18 months	23.1	43.7	31.7	3.4
	24 months	15.7	36.3	25.6	3.4
%TWL	6 months	12.5	30.7	19.5	3.4
	12 months	25.8	47.5	35.9	4.9
	18 months	31.1	57.2	42.9	4.9
	24 months	35.1	62.8	48	5
DM (HbA1C)	6 months	5.1	8.5	6.7	0.8
	12 months	4.4	7.9	6	0.8
	18 months	4.7	7.4	5.5	0.8
	24 months	5.1	6.1	5.5	0.2
Hb (12–16 gm%)	6 months	11.1	14.3	12.7	0.8
	12 months	10.7	13.9	12.2	0.8
	18 months	10.3	13.5	11.9	0.8
	24 months	9.7	13.2	11.4	0.9
Fe (60–180 mcg/dl)	6 months	97	147	122	10
	12 months	78	130	105.7	10.8
	18 months	92	123	107.5	9.6
	24 months	82	116	98.9	9.7
Ca (8.6–10.6 mg/dl)	6 months	8.7	9.8	9.2	0.3
	12 months	8.4	9.5	8.9	0.3
	18 months	8.6	9.3	8.9	0.2
	24 months	8.5	9.1	8.7	0.1
Albumin (3.5–4.5 gm%)	6 months	3.6	4.3	3.9	0.2
	12 months	3.4	4.2	3.8	0.2
	18 months	3.3	4.1	3.6	0.2
	24 months	3.3	3.9	3.6	0.2
Vit D (20–50 ng/ml)	6 months	25	42	33.8	5.1
	12 months	22	39	30.3	4.8
	18 months	20	35	27.5	4.3
	24 months	17	31	24.2	4.1
PTH (10–55 pg/ml)	6 months	42	54	48	3.8
	12 months	46	66	56.6	6
	18 months	52	72	62	5.8
	24 months	54	88	69.9	10.3
		Incidence		*N*	
Alkaline reflux	6 months	No		198	
		Yes		16	
	12 months	No		201	
		Yes		13	
	18 months	No		201	
		Yes		13	
	24 months	No		206	
		Yes		8	
HTN	6 months	No		120	
		Yes		94	
	12 months	No		195	
		Yes		19	
	18 months	No		201	
		Yes		13	
	24 months	No		211	
		Yes		3	

^*^Skewness value was between 1 and − 1 so mean ± SD was used

### Weight, BMI, and %TWL

**Fig. 1:**
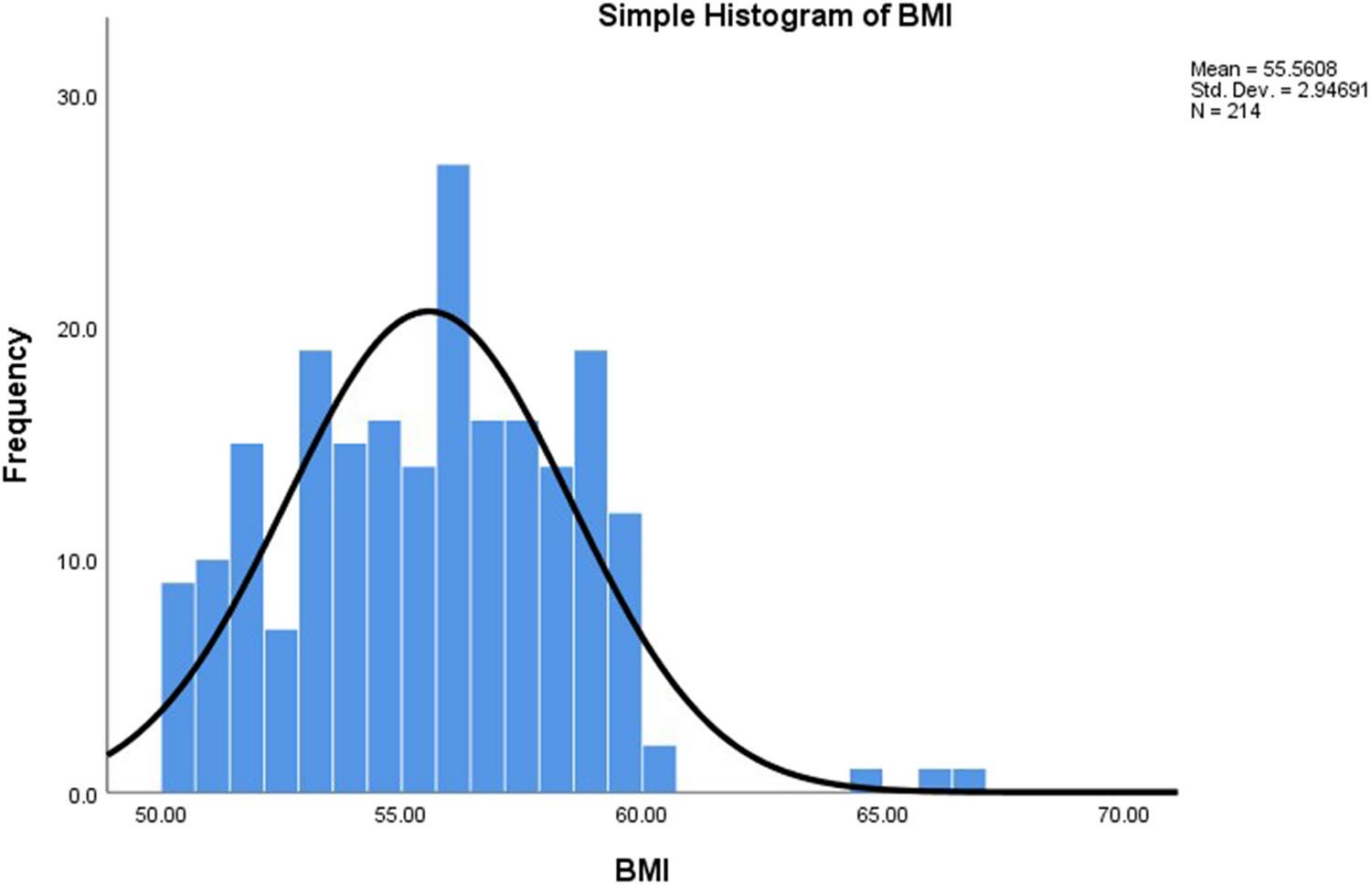
Histogram of BMI preoperatively (normal distribution)

**Fig. 2:**
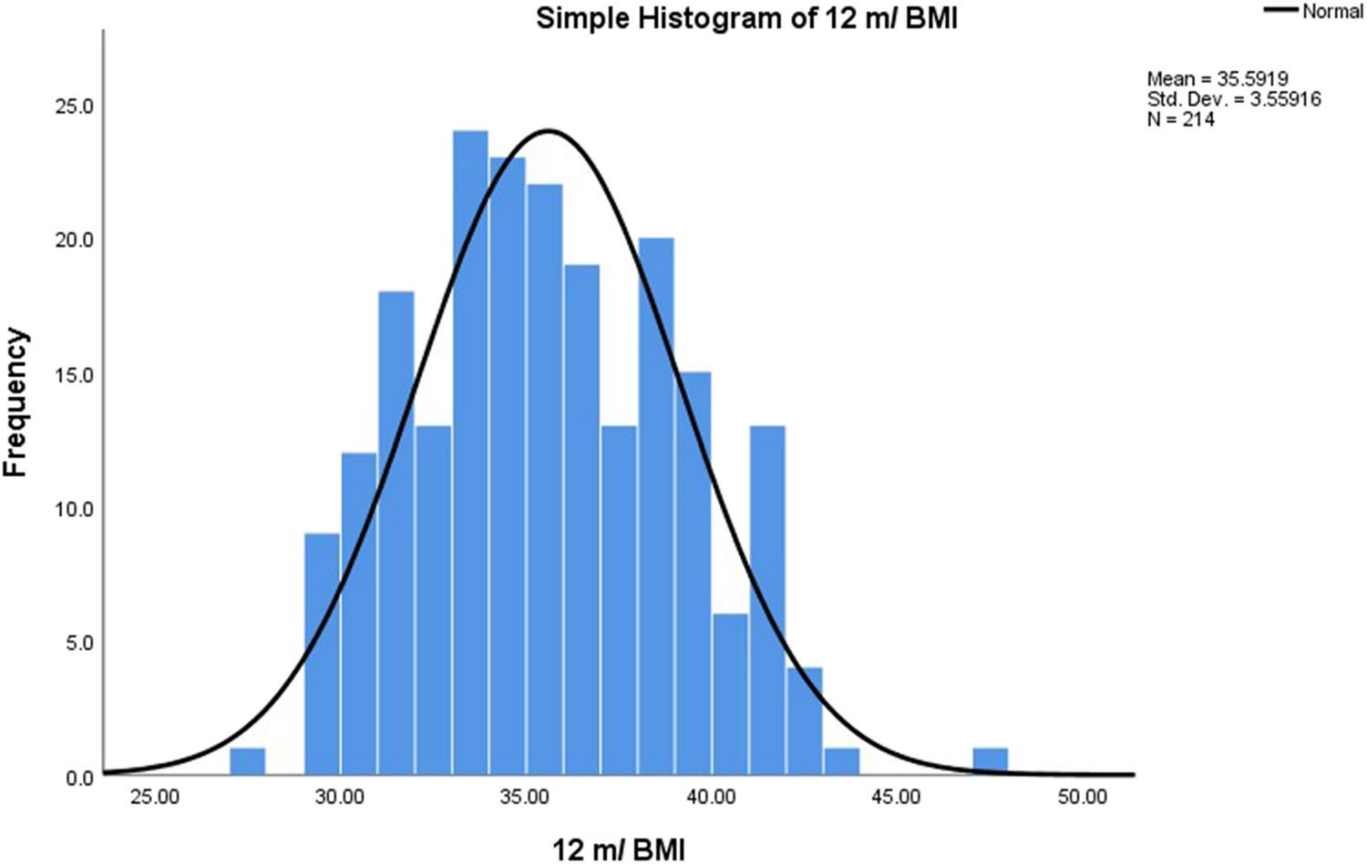
Histogram of BMI at 12 months (normal distribution)

**Fig. 3:**
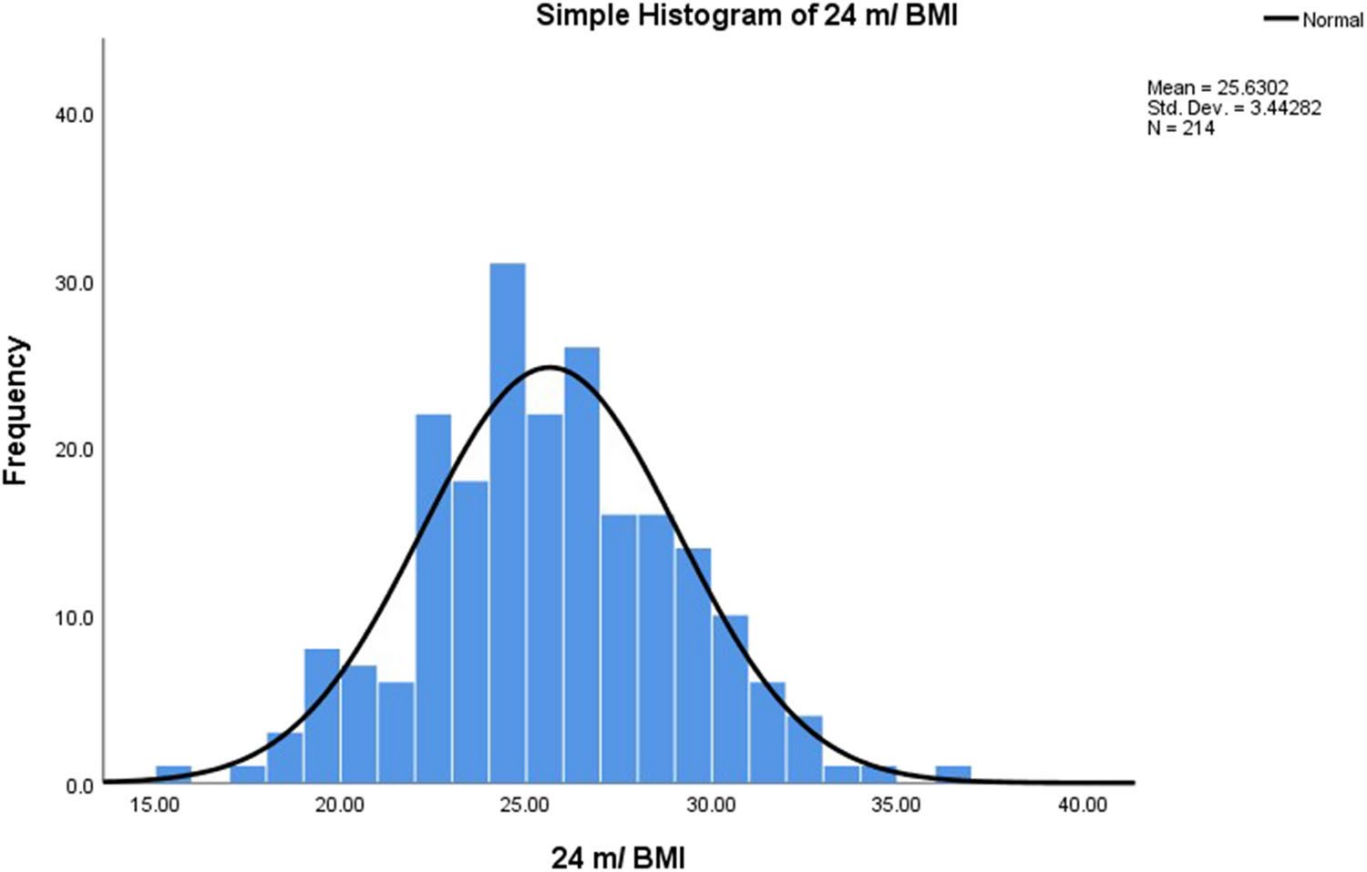
Histogram of BMI at 24 months (normal distribution)

The weight and BMI mean ± SD showed highly significant differences with time from preoperative value till 24 months postoperative by repeated measure ANOVA (*p* < 0.001) with highly significant differences, and positive correlation within each pair of successive readings by paired *t* test till 24 months postoperative (71.5 ± 10.8 kg and 25.6 ± 3.4 kg/m^2^ at 24 months respectively) (Tables 3 and 4). A histogram graphic representation of BMI preoperatively, at 12 months and 24 months, showed normal distribution of values (skewness value between 1 and − 1 with standard error 0.166) with most subjects distributed around BMI 25 kg/m^2^ denoting achievement of target BMI as seen in (Figs. 1, 2, and 3).

**Table 3:** Repeated measure ANOVA of weight, BMI, %TWL, level of HbA1C, Hb, Fe, Ca, Alb, Vit D, and PTH

	Mauchly’s test of sphericity	ANOVA *F* statistic	ANOVA *p* value^*^	ηρ^2**^
Weight	*X*^2^ = 541.3 *p* < 0.001^*^	*F* = 15,846	< 0.001	0.987
BMI	*X*^2^ = 620.3 *p* < 0.001^*^	*F* = 12,911.7	< 0.001	0.984
%TWL	*X*^2^ = 349.9 *p* < 0.001^*^	*F* = 7692.6	< 0.001	0.973
DM (HbA1C)	*X*^2^ = 1177.9 *p* < 0.001^*^	*F* = 1030.4	< 0.001	0.829
Hb (12–16 gm%)	*X*^2^ = 877.6 *p* < 0.001^*^	*F* = 2670.9	< 0.001	0.926
Fe (60–180 mcg/dl)	*X*^2^ = 491.6 *p* < 0.001^*^	*F* = 1918.6	< 0.001	0.900
Ca (8.6–10.6 mg/dl)	*X*^2^ = 219.5 *p* < 0.001^*^	*F* = 153.6	< 0.001	0.419
Alb (3.5–4.5 gm%)	*X*^2^ = 20.4 *p* = 0.015^*^	*F* = 211.8	< 0.001	0.499
Vit D (20–50 ng/ml)	*X*^2^ = 13.2 *p* = 0.154	*F* = 330.2	< 0.001	0.608
PTH (10–55 pg/ml)	*X*^2^ = 126.1 *p* < 0.001^*^	*F* = 663.7	< 0.001	0.757

^*^Greenhouse–Geisser correction was applied as Mauchly’s assumption of sphericity was violated (*p* < 0.05)

^**^ηρ^2^ (partial eta squared) for effect size. Observed power was 1

**Table 4:** Paired *t* test with correlation for each pair of recorded values in BMI, %TWL, level of HbA1C, Hb, Fe, Ca, Alb, Vit D, PTH, alkaline reflux incidence, and HTN

	Pair	Correlation			Paired *t* test	
		Coefficient	*p* value	Direction	*t*	*p* value
Weight	Preop. − 6 m	0.874	0.000	Positive	87.1	0.000
	6 m–12 m	0.920	0.000	Positive	83.9	0.000
	12 m–18 m	0.968	0.000	Positive	55.7	0.000
	18 m–24 m	0.972	0.000	Positive	95	0.000
BMI	Preop. − 6 m	0.801	0.000	Positive	80.9	0.000
	6 m–12 m	0.888	0.000	Positive	81.4	0.000
	12 m–18 m	0.957	0.000	Positive	54.4	0.000
	18 m–24 m	0.958	0.000	Positive	88.6	0.000
%TWL	6 m–12 m	0.811	0.000	Positive	− 81.9	0.000
	12 m–18 m	0.928	0.000	Positive	− 54.5	0.000
	18 m–24 m	0.942	0.000	Positive	− 43.9	0.000
DM (HbA1C)	Preop. − 6 m	0.985	0.000	Positive	102.1	0.000
	6 m–12 m	0.976	0.000	Positive	52.9	0.000
	12 m–18 m	0.974	0.000	Positive	39.0	0.000
	18 m–24 m	− 0.021	0.765	Negative	− 0.7	0.432
Hb	Preop.- 6 m	0.975	0.000	Positive	44.8	0.000
	6 m–12 m	0.984	0.000	Positive	42.3	0.000
	12 m–18 m	0.981	0.000	Positive	33.3	0.000
	18 m–24 m	0.987	0.000	Positive	45.9	0.000
Fe	Preop. − 6 m	0.865	0.000	Positive	110.7	0.000
	6 m–12 m	0.904	0.000	Positive	51.1	0.000
	12 m–18 m	−0.046	0.505	Negative	− 1.7	0.078
	18 m–24 m	0.129	0.060	Positive	9.8	0.000
Ca	Preop. − 6 m	0.021	0.759	Positive	6.4	0.000
	6 m–12 m	− 0.052	0.449	Negative	9.2	0.000
	12 m–18 m	0.015	0.833	Positive	1	0.292
	18 m–24 m	0.062	0.364	Positive	6.7	0.000
Albumin	Preop. − 6 m	0.124	0.070	Positive	10.2	0.000
	6 m–12 m	0.126	0.067	Positive	7.0	0.000
	12 m–18 m	− 0.053	0.441	Negative	5.3	0.000
	18 m–24 m	0.087	0.205	Positive	2.8	0.005
Vit D	Preop. − 6 m	0.051	0.460	Positive	11.2	0.000
	6 m–12 m	0.079	0.249	Positive	7.4	0.000
	12 m–18 m	0.007	0.921	Positive	6.2	0.000
	18 m–24 m	− 0.024	0.724	Negative	8	0.000
PTH	Preop. − 6 m	− 0.027	0.698	Negative	− 18	0.000
	6 m–12 m	0.010	0.883	Positive	− 17.4	0.000
	12 m–18 m	0.040	0.557	Positive	− 9.6	0.000
	18 m–24 m	0.110	0.110	Positive	− 10.1	0.000
Alkaline reflux^*^	6 m–12 m				Fisher exact test	0.000
	12 m–18 m				Fisher exact test	0.000
	18 m–24 m				Fisher exact test	0.000
HTN^**^	Preop. − 6 m				*x*^*2*^ = 1.2	0.255
	6 m–12 m				*x*^*2*^ = 26.6	0.000
	12 m–18 m				Fisher exact test	0.000
	18 m–24 m				Fisher exact test	0.000

^*^Fisher exact test was used. ^**^Fisher exact test and Pearson’s chi square test were used

The %TWL mean ± SD showed highly significant differences with time from 6 months value till 24 months postoperative by repeated measure ANOVA (*p* < 0.001) with highly significant differences and positive correlation within each pair of successive readings by paired *t* test till 24 months postoperative (48 ± 5% at 24 months) (Tables 3 and 4).

**Fig. 4:**
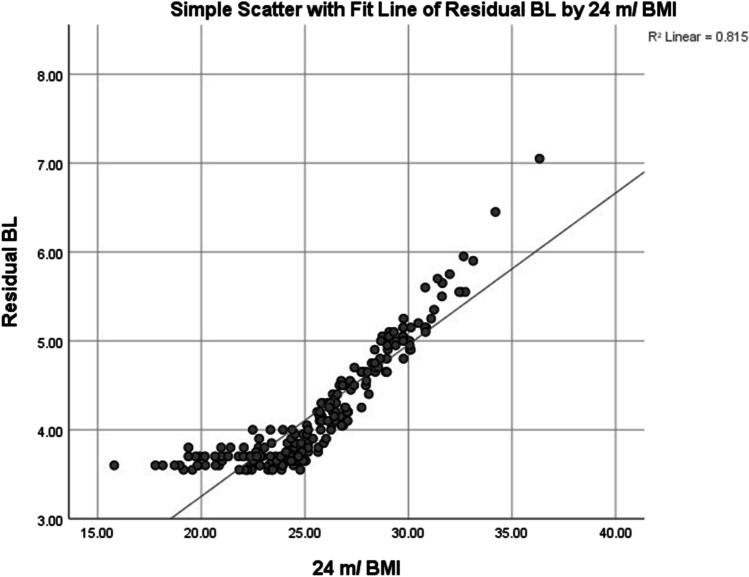
Scatter plot with linear fit of RBL against 24-month BMI

**Fig. 5:**
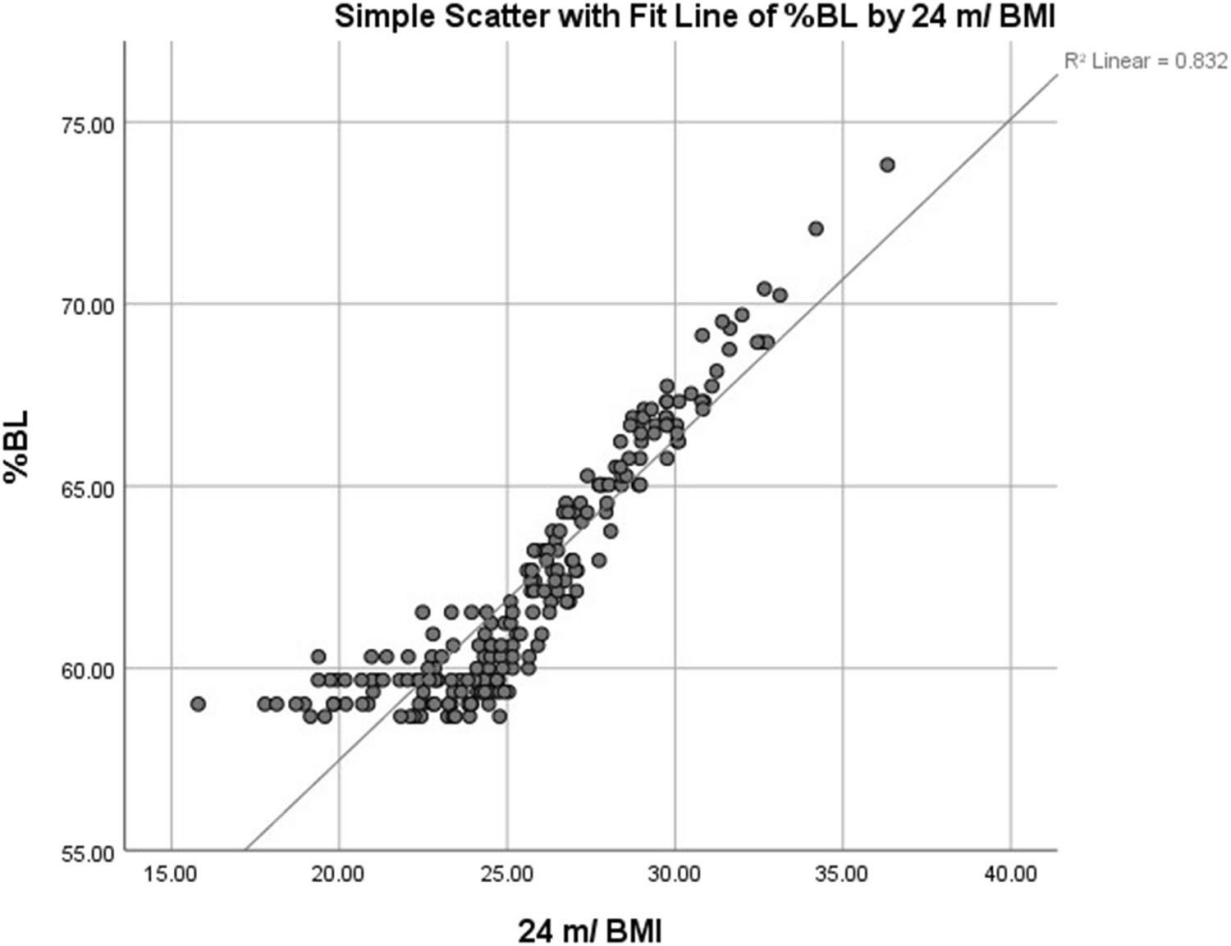
Scatter plot with linear fit of %BL against 24-month BMI

A Pearson product-moment bivariate correlation was run to determine the relationship between the RBL and 24-month BMI. There was a strong positive correlation between the RBL and 24-month BMI which was statistically significant (*r* = 0.903, *n* = 214, *p* < 0.001). Scatter plot with linear fit of RBL against 24-month BMI was shown in (Fig. 4) with *R*^2^ = 0.815. A Pearson product-moment bivariate correlation was run to determine the relationship between the %BL and 24-month BMI. There was a strong positive correlation between the %BL and 24-month BMI which was statistically significant (*r* = 0.912, *n* = 214, *p* < 0.001). Scatter plot with linear fit of %BL against 24-month BMI was shown in (Fig. 5) with *R*^2^ = 0.832.

A RBL of 4.2 ± 0.65 m was enough to achieve and maintain the target BMI evidenced by the strong positive correlation between RBL and 24-month BMI. The application of nearly 40:60% ratio between the bypassed length and residual length allowed for achieving target BMI in this study.

### DM and HTN

The level of HbA1C (mean ± SD) showed highly significant differences with time from preoperative value till 24 months of postoperative repeated measure ANOVA (*p* < 0.001) with highly significant differences and positive correlation within each pair of successive readings by paired *t* test till 18 months postoperative and negative correlation and non-significant difference (*p* = 0.432) within 18–24 months pair (5.5 ± 0.8 gm% at 18 months and 5.5 ± 0.2 gm% at 24 months) (Tables 3 and 4).

The HTN incidence showed a non-significant decrease from preoperative value till 6 months postoperative and a highly significant decrease from 6 to 12, 12 to 8, and 18 to 24 months (only 1.4% at 24 months) (Tables 1, 2, and 4).

### Nutritional Status

The level of Hb, Fe, Ca, albumin, vitamin D, and PTH (mean ± SD) showed highly significant differences with time from preoperative value till 24 months postoperative by repeated measure ANOVA (*p* < 0.001) (Table 3).

The level of Hb (mean ± SD) showed highly significant differences with positive correlation within each pair of successive readings by paired *t* test till 24 months postoperative (11.4 ± 0.9 gm% at 24 months) (Tables 2 and 4). The level of Fe (mean ± SD) showed highly significant differences with positive correlation within each pair of successive readings by paired *t* test (at preop. − 6 months, 6–12 months, and 18–24 months with 98.9 ± 9.7 mcg/dl at 24 months) and negative correlation with significant difference (*p* = 0.078) at 12–18 months (Tables 2 and 4).

The level of Ca (mean ± SD) showed highly significant differences with positive correlation within each pair of successive readings by paired *t* test (at preop. − 6 months and 18–24 months with 8.7 ± 0.19 gm/dl at 24 months), negative correlation with a highly significant difference at 6–12 months, and positive correlation with a non-significant difference (*p* = 0.292) at 12–18 months (Tables 2 and 4). The level of albumin (mean ± SD) showed highly significant differences with positive correlation within each pair of successive readings by paired *t* test (at preop. − 6 months, 6–12 months, and 18–24 months with 3.6 ± 0.2 gm/dl at 24 months) and negative correlation with a highly significant difference at 12–18 months (Tables 2 and 4).

The level of vitamin D (mean ± SD) showed highly significant differences with positive correlation within each pair of successive readings by paired *t* test (at preop. − 6 months, 6–12 months, and 12–18 months) and negative correlation with a highly significant difference at 18–24 months (24.2 ± 4.1 ng/ml at 24 months) (Tables 2 and 4).

The level of PTH (mean ± SD) showed highly significant differences with positive correlation within each pair of successive readings by paired *t* test (at 6–12 months, 12–18 months, and 18–24 months with 69.9 ± 10.3 pg/ml at 24 months) and negative correlation with a highly significant difference at preop. − 6 months (Tables 2 and 4).

The effect size expressed by partial eta squared (ηρ^2^) ranged from 0.419 to 0.987 by repeated measure ANOVA yielding observed power 1.00.

### Alkaline Reflux

The alkaline reflux incidence showed a highly significant decrease from 6 to 12, 12 to 18, and 18 to 24 months (only 3.7% at 24 months) (Tables 1, 2, and 4).

## Discussion

OAGB had achieved its place as one of the popular bariatric procedures. However, the potentiality of the classic OAGB with 180-cm biliopancreatic limb (BPL) to achieve weight loss decreases with an increase in BMI. The YOMEGA study compared the 200-cm BPL OAGB with RYGB in patients with BMI < 50 kg/m^2^ and found no significant difference between them [11]. Yet, the patients with BMI > 50 kg/m^2^ will not achieve the target BMI with 180–200-cm BPL especially with long total bowel length (TBL), and many bariatric surgeons resort to long BPL RYGB. This study included patients with BMI > 50 kg/m^2^ who underwent 250-cm BPL OAGB and had TBL > 6 m to evaluate the effects and outcomes in those patients.

The procedure of OAGB adopted in this study was to do gastrojejunostomy at a distance of 250 cm from the DJ junction after creating a gastric pouch of 15 cm long on a 36 F bougie. A study by Plamper et al. [12] performed OAGB at a length of 250 cm for patients of BMI 50–60 while Madhok et al. [13] performed OAGB at 200 cm and both with good results. In this study, the procedure included measuring the TBL to ensure the presence of at least 350 cm distally to prevent the incidence of short common limb (CL) and decrease the nutritional deficiencies. Similar lengths of CL were suggested by Khalaj et al., Felsenreich et al., and Soong et al. [14–16]. OAGB had a short operative time with similar results obtained by Soong et al. and Lee et al. [16, 17].

As regards the weight, BMI, and %TWL preoperatively and at 6, 12, 18, and 24 months postoperatively, there was a significant decrease in weight and BMI from preoperative values to the postoperative values till 24 months in OAGB with a mean BMI of around 25 kg/m^2^ which was the target BMI (20–25 kg/m^2^). As regards the %TWL, OAGB achieved 48 ± 5%. A study by Singla et al. [18] reported a %TWL of 39.9% with OAGB in super obesity which was less than this study. Another study done by Bhandari et al. [3] stated that %TWL at 2 years was 43% in OAGB and also in a study by Soong et al. [16].

A RBL of 4.2 ± 0.65 m was enough to achieve and maintain the target BMI evidenced by the strong positive correlation between RBL and 24 months BMI. The application of nearly 40:60% ratio between the bypassed length and residual length allows for achieving target BMI without serious consequences in patients with > 6 m TBL.

As regards the HTN, there was a decrease in the percent of patients suffering from HTN from 61.7% preoperatively to 1.7% at 24 months as the results obtained by Magouliotis et al. [19]. The HTN status did not significantly change in the first 6 months then showed progressive improvement from 6 to 24 months. As regards the DM, there was a reduction in the mean level of HbA1C postoperatively to non-diabetic levels (< 6 gm%) at 18 and remained almost stationary till 24 months. This confirms the metabolic effect in remission of HTN and DM of OAGB.

The incidence of alkaline reflux was 7.5% at 6 months that was reduced to 3.7% at 24 months. In literature, alkaline reflux varied from 0.6 to 10% [20]. The adopted method of the long gastric pouch as in the study by Soong et al. and Deitel et al. [16, 21] and the application of draining technique in the form of side-to-side anastomosis of gastrojejunostomy and fixation of afferent and efferent loops to help passage of gastric contents distally lead to a relatively low incidence of alkaline reflux in comparison to other studies as that performed by Keleidari et al. [22].

The levels of Hb, Fe, Ca, and vitamin D levels showed progressive decrease till 24 months but remain within normal range. The albumin levels decrease till 18 months then showed a tendency to stabilize by 24 months. The PTH level remains high normal till 12 months then became higher than the normal range with a tendency to rise till 24 months.

OAGB had decreased nutrient absorption owing to the bypassed part of the proximal jejunum. As the long BP OAGB had 250 cm of excluded jejunum, nutrients absorbed in the proximal jejunum had more deficiencies in OAGB as the results confirmed in the reduction of Hb, Fe, Ca, albumin, and Vit D levels with significant differences with time despite the compliance of the patients to postoperative vitamin and mineral supplements, yet the levels are within the normal range. PTH levels showed a significant increase with time yet were mildly elevated after 12 months due to chronically impaired absorption of Ca.

Similar results concerning Hb, Alb, Ca, and PTH were obtained by Soong et al. [16] and Omar et al. [23]. Similar differences between OAGB and RYGB concerning low Hb levels were obtained by Lee et al. [17]. Carbajo et al. [24] stated that in OAGB nutritional deficiencies and malnutrition are increasingly reported when the bypassed jejunum is > 250 cm. Charalampos et al. [25] did not find any differences in nutritional deficiencies in OAGB between BP limbs of 200, 250, and 300 cm. IFSO Consensus Conference recommends OAGB to be performed if BMI > 50 with the presence of a suitable length common channel in OAGB if BP is to be more than 200 cm [26].

There is a controversy on the length of bypassed jejunum ranging from 150 to 350 cm in OAGB [14, 27]. This study concluded that OAGB with 250 cm bypassed part of jejunum (with nearly 40:60% ratio between the bypassed part and the remaining part) is a good choice for patients with BMI > 50 kg/m^2^ [16] after ensuring the presence of at least 350 cm distally as CL (it was 4.2 ± 0.65 m in this study) with comparable results with long BP RYGB (BP limb = 150 cm and AL = 100 cm) with < 30 BMI kg/m^2^ after 2 years and more than 45% TWL. Both procedures have good metabolic effects on associated comorbidities with remission of HTN and DM. OAGB had a more yet non-significant incidence of alkaline reflux due to long gastric pouch (15 cm) and drainage procedure through fixation of afferent and efferent limbs and if present can be controlled by medications up to 50% of cases. RYGB had significantly more operative time as confirmed by Soong et al. [16] with more incidence of complications.

## Conclusion

Two-hundred fifty-cm BPL OAGB with > 350-cm common limb in patients with BMI > 50 kg/m^2^ and total bowel length more than 6 m can achieve a target BMI of 25 kg/m^2^ with nearly 40:60% ratio between the bypassed length and residual length. This was accompanied by remission of associated comorbidities as HTN and DM without causing serious nutritional deficiencies with the maintenance of target BMI for at least 2 years. The limitations of this study were the absence of long-term follow-up till 5–10 years, the absence of comparative design, and the need for larger number of patients.
